# 2D-QSAR and 3D-QSAR Analyses for EGFR Inhibitors

**DOI:** 10.1155/2017/4649191

**Published:** 2017-05-29

**Authors:** Manman Zhao, Lin Wang, Linfeng Zheng, Mengying Zhang, Chun Qiu, Yuhui Zhang, Dongshu Du, Bing Niu

**Affiliations:** ^1^Shanghai Key Laboratory of Bio-Energy Crops, College of Life Science and Shanghai University High Performance Computing Center, Shanghai University, Shanghai 200444, China; ^2^Department of Oncology, Hainan General Hospital, Haikou, Hainan 570311, China; ^3^Department of Radiology, Shanghai General Hospital, Shanghai Jiao Tong University School of Medicine, Shanghai 200080, China; ^4^Changhai Hospital, Second Military Medical University, Shanghai 200433, China; ^5^Department of Life Science, Heze University, Heze, Shandong 274500, China

## Abstract

Epidermal growth factor receptor (EGFR) is an important target for cancer therapy. In this study, EGFR inhibitors were investigated to build a two-dimensional quantitative structure-activity relationship (2D-QSAR) model and a three-dimensional quantitative structure-activity relationship (3D-QSAR) model. In the 2D-QSAR model, the support vector machine (SVM) classifier combined with the feature selection method was applied to predict whether a compound was an EGFR inhibitor. As a result, the prediction accuracy of the 2D-QSAR model was 98.99% by using tenfold cross-validation test and 97.67% by using independent set test. Then, in the 3D-QSAR model, the model with *q*^2^ = 0.565 (cross-validated correlation coefficient) and *r*^2^ = 0.888 (non-cross-validated correlation coefficient) was built to predict the activity of EGFR inhibitors. The mean absolute error (MAE) of the training set and test set was 0.308 log units and 0.526 log units, respectively. In addition, molecular docking was also employed to investigate the interaction between EGFR inhibitors and EGFR.

## 1. Introduction

Epidermal growth factor receptor (EGFR), a transmembrane glycoprotein, is classified to the prototype of receptor tyrosine kinases (TKs) family that includes EGFR, ErbB-2, ErbB-3, and ErbB-4. EGFR is activated by its cognate ligands via forming a homodimer or heterodimer with other members of the EGFR family, such as epidermal growth factor (EGF) and transforming growth factor alpha (TGF-*α*) [1]. Several signal transduction cascades are initiated when EGFR is activated and then lead to DNA synthesis and cell proliferation [2, 3]. While EGFR is amplified or mutated, DNA synthesis and cell proliferation will be abnormal and lead to cancer. Currently, the amplification or mutation of EGFR has been found in human solid tumors, such as glioma, lung cancer, ovarian cancer, and breast cancer. Hence, EGFR is also considered to be a potential anticancer target in this disease [4–8]. Many EGFR inhibitors have been developed and approved by the FDA, such as lapatinib, which has been applied for the treatment of breast cancer [9]. Moreover, other EGFR inhibitors like temozolomide, lomustine, erlotinib, and gefitinib, are approved by the FDA for the treatment of glioma [10, 11]. However, the existing EGFR inhibitors are beyond people's expectation due to selectivity, toxicity, and side effect. Hence, it is necessary to design and synthesize new potential EGFR inhibitors.

Quantitative structure-activity relationship (QSAR) was a valuable tool for many different applications, including drug discovery, predictive toxicology, and risk assessment [12–14]. The applicability domain of QSAR models, defined by the Organization for Economic Co-operation and Development (OECD) according to Principle 3, includes the physicochemical, the structural, and the biological domain [15–17]. Initially, two-dimensional quantitative structure-activity relationship (2D-QSAR) was widely explored and used in medicinal chemistry study. However, some limitations spurred the appearance of three-dimensional quantitative structure-activity relationship (3D-QSAR). In the 3D-QSAR study, the correlation between 3D steric and electrostatic fields and biologically activity draws attention. For the molecular field study, CoMFA was widely used preliminarily. However, the time-consuming limit stimulates the advent of TopCoMFA. TopCoMFA overcomes the weakness and uses an objective method to fragment and align the molecules. In addition, the fragmentation process is automated except for some specific bonds that should be cleaved manually. Of course, TopCoMFA and CoMFA also have similarity that they both share QSAR PLS analysis. The details about TopCoMFA and CoMFA are in [18].

Drug development is a long process, and it requires a vast amount of material and financial resources. QSAR and molecular docking technology have been extensively employed in drug virtual screening and potential molecular targets prediction, which may shorten the cycle of the drug development [19–22]. In this work, 2D-QSAR model was employed to determine EGFR inhibitor, and the 3D-QSAR model was used to predict the activity. Finally, molecular docking was applied to investigate the binding sites.

## 2. Materials and Methods

### 2.1. CfsSubsetEval Method and Greedy Stepwise Algorithm

A data set containing *n* vectors has 2^*n*^ possible combinations of features for the subset. A useful subset which can correctly predict other compounds is one of 2^*n*^ combinations. The best way to find an optimal subset is to try all the possible feature combinations. However, this strategy is difficult to carry out due to the huge computation. In this study, the CfsSubsetEval (CFS) search method combined with Greedy Stepwise (GS) algorithm was employed to search the optimal feature subset. The main idea of the GS algorithms is to make the best choice when selecting good features. The CFS method was used to evaluate the attribute. Thus, the CFS method, combined with the GS algorithm, was employed to select the optimal subset from these 2^*n*^ combinations. Additional details about the CFS method and the GS algorithm could be found in [23–25].

### 2.2. SVM

Support vector machine (SVM), a supervised learning algorithm, is usually used for pattern recognition classification [26]. SVM was employed for the classification and sensitivity analysis in our study due to its high performance in many studies [25, 27, 28].

### 2.3. Topomer CoMFA

Topomer CoMFA, possessing both the topomer technique and CoMFA technology, can overcome the alignment problem of CoMFA [18, 29]. Partial least squares (PLS) regression is employed to build the topomer CoMFA model, and the leave-one-out (LOO) cross-validation is used to evaluate the model. Additional details about the topomer CoMFA can be found in [29–31].

### 2.4. Data Preparation

100 inhibitors derived from the literature and 185 noninhibitors downloaded from the DUD database (http://dud.docking.org) were collected [32–41]. For 2D-QSAR study, the data set containing inhibitors and noninhibitors was randomly divided into three training sets which accounted for 75%, 70%, and 50% of the whole data set, respectively (see Supplementary Material 1, available online at https://doi.org/10.1155/2017/4649191). For 3D-QSAR study, the 100 inhibitors were randomly divided into a training set (77 molecules) and an independent test set (23 molecules).

### 2.5. Molecular Descriptor Calculation

Molecular descriptor can reflect physicochemical and geometric properties of the compounds. In this study, forty-five molecular descriptors calculated by the ChemOffice were applied to represent compounds [42]. First, three-dimensional structures of the molecules were optimized by MM+ force field with the Polak-Ribiere algorithm until the root-mean-square gradient became less than 0.1 Kcal/mol. Then, quantum chemical parameters were obtained for the most stable conformation of each molecule by using PM3 semiempirical molecular orbital method at the restricted Hartree-Fock level with no configuration interaction.

### 2.6. Validation Methods for Prediction Results

In this study, tenfold cross-validation test and independent set test were applied to evaluate the prediction ability of the 2D-QSAR model. For the tenfold cross-validation test, the data set was divided into ten subsets. Nine subsets were used as the training set and the left subset was predicted. In turn, each subset was omitted in order to be predicted, and the correct rate was obtained from each trial. The average of the correct rate from ten trials was used to estimate the accuracy of the algorithm [43–45].

### 2.7. Prediction Measurement

Sensitivity (SN), specificity (SP), overall accuracy (ACC), and Matthew's correlation coefficient (MCC) were employed to evaluate the 2D prediction model. The SN, SP, ACC, and MCC can be represented as

(1) SN=TPTP+FN,SP=TNTN+FP,ACC=TP+TNTP+TN+FP+FN,MCC=TP×TN−FP×FNTN+FN×TN+FP×TP+FN×TP+FP.

TP, TN, FP, and FN are true positives, true negatives, false positives, and false negatives, respectively.

In the topomer CoMFA model, *q*^2^, *r*^2^, and MAE were applied to evaluate the model [46]. The cut-off value of *q*^2^ is 0.5. The MAE of the test set was less than 0.1 × training set range and MAE + 3 × *σ* according to the MAE based criteria. The optimized model was determined by the highest *q*^2^, and the validity of the model depends on *r*^2^ value [47].

### 2.8. Steric and Electrostatic Field Analysis

Topomer CoMFA analysis is an effective approach which has been applied in drug design for HIV, central nervous system diseases, and other tumors [48–50]. In the topomer CoMFA model, there are two different ways to calculate the molecular field. One way is to reduce the field contributions of fragmenting atoms; the other way is to calculate the steric and electrostatic fields on a regularly spaced grid. For detailed information, see [51]. Topomer CoMFA analysis is used to calculate the steric field and electrostatic fields of R1 and R2 groups. Steric and electrostatic field analysis may help design novel EGFR drugs.

### 2.9. Molecular Docking

SYBYL X-2.0 was used for molecular docking based on its Surflex-Dock module [52]. The crystal structure of EGFR with the resolution of 2.6 Å was downloaded from the Protein Data Bank (PDB ID: 1M17) [53]. Protein was prepared with protein structure preparation module of the SYBYL X-2.0. All the water molecules and ligands were deleted, and hydrogen atoms were added to the crystal structure. In addition, positive and negative charges were added to N-terminal and C-terminal regions of the EGFR which became NH^3+^ and COO^−^. EGFR inhibitors were minimized at physiological pH 7.0 with hydrogen atoms and charge by using Powell energy gradient method and the Gasteiger-Huckel system.

## 3. Results

### 3.1. Feature Selection and the 2D-QSAR Prediction Model

**Figure 1:**
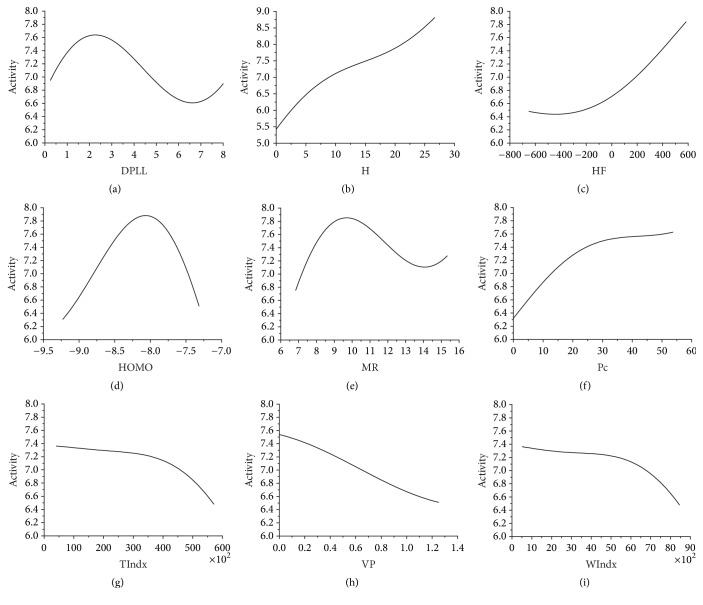
(a) Activity value versus DPLL. (b) Activity value versus H. (c) Activity value versus HF. (d) Activity value versus HOMO. (e) Activity value versus MR. (f) Activity value versus Pc. (g) Activity value versus TIndx. (h) Activity value versus VP. (i) Activity value versus WIndx.

A feature subset containing nine molecular descriptors (DPLL, H, HF, HOMO, MR, Pc, TIndx, VP, and WIndx) was obtained based on CFS combined with GS algorithms. Sensitivity analysis was applied to these nine descriptors to evaluate how they affected the activity of EGFR inhibitors (see Figure 1).

Based on the optimal features subset, the SVM classifier method was used to build the 2D-QSAR prediction model. As a result, the prediction accuracy of these models whose data set accounted for 75%, 70%, and 50% of the whole data set was 98.13%, 98.99%, and 91.24%, respectively, by tenfold cross-validation test. The sensitivity, specificity, and overall accuracy of these three models were more than 90%, which indicated that changing the size of the training set had a little impact on the quality of the 2D-SAR models (see Table 1). The model built via the data set accounting for 70% of the whole data set was chosen finally due to its higher prediction accuracy and smaller size. Although the result of the tenfold cross-validation test was well, it was not good enough for evaluating the classifier as the SVM classifier might be overfitted. To validate the reliability of the classifier, an independent test set was further employed in this study. As a result, the prediction accuracy of the independent set test was 97.67%.

### 3.2. 3D-QSAR Prediction Model

The training set was employed to build the topomer CoMFA model by fragmenting EGFR inhibitors into R1 and R2 groups. Two topomer CoMFA models were generated by two cutting ways. The topomer CoMFA model 2 with higher *q*^2^ and *r*^2^ values was selected to analyze and predict EGFR inhibitors' activities (see Table 2).

**Figure 2:**
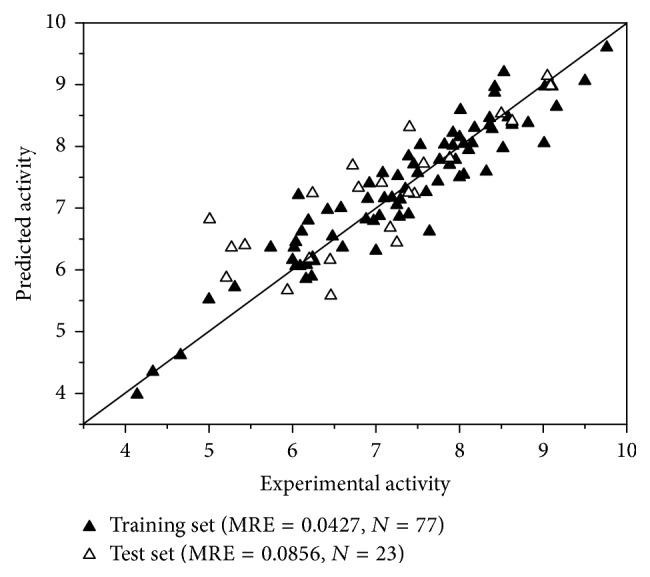
Scatterplot of experimental data versus predicted data from topomer CoMFA model 2.

The experimental and predicted activities of the training set and the independent test set were listed in Table 3 and Figure 2. As a result, the MAE and *r*^2^ of the training set were 0.308 and 0.888, respectively. The training set range was 7.32. To estimate the reliability of model 2, the independent set test was used to evaluate the model. The MAE and *r*^2^ of the test set were 0.526 and 0.681, respectively. The MAE of the test set was less than 0.732 (0.1 × training set range) and 1.903 (MAE_(training set)_ + 3 × *σ*).

**Figure 3:**
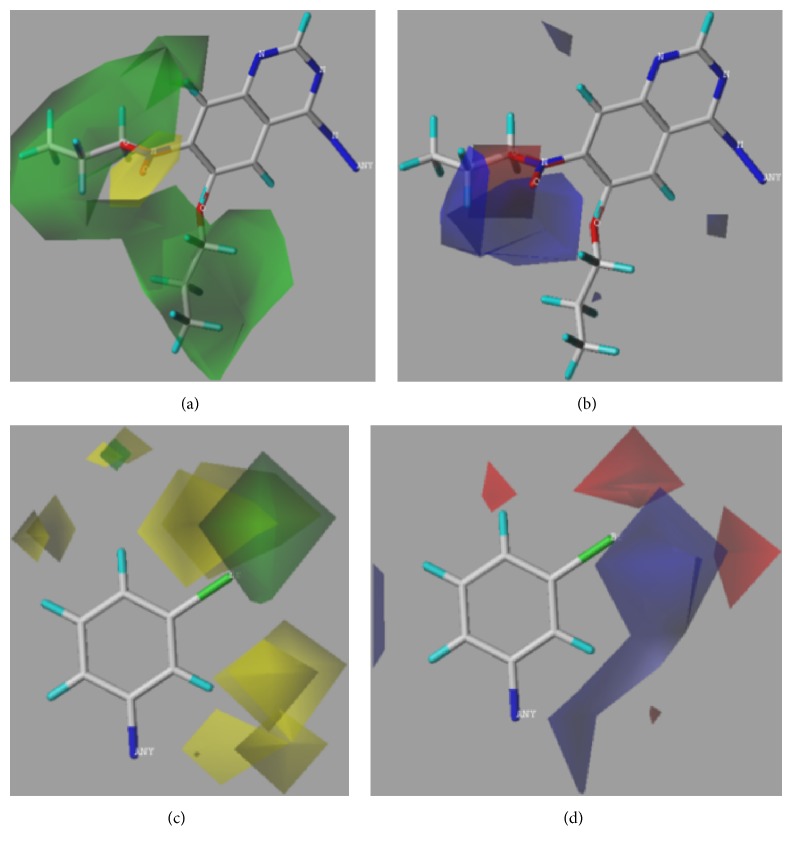
3D contour maps of topomer CoMFA model for R1 and R2 of compound** 33**. (a) and (c) present steric contour map. (b) and (d) present electrostatic field map. Green, yellow, blue, and red represent large volume, small volume, positively charged, and negatively charged groups, respectively.

Additionally, steric and electrostatic contour maps of R1 and R2 groups were obtained. Compound** 33** was selected to study how to redesign EGFR inhibitors due to the highly activity (see Figure 3). From Figure 3, it could be concluded that large volume and positively charged groups were added, which can increase compound activity.

### 3.3. Molecular Docking

**Figure 4:**
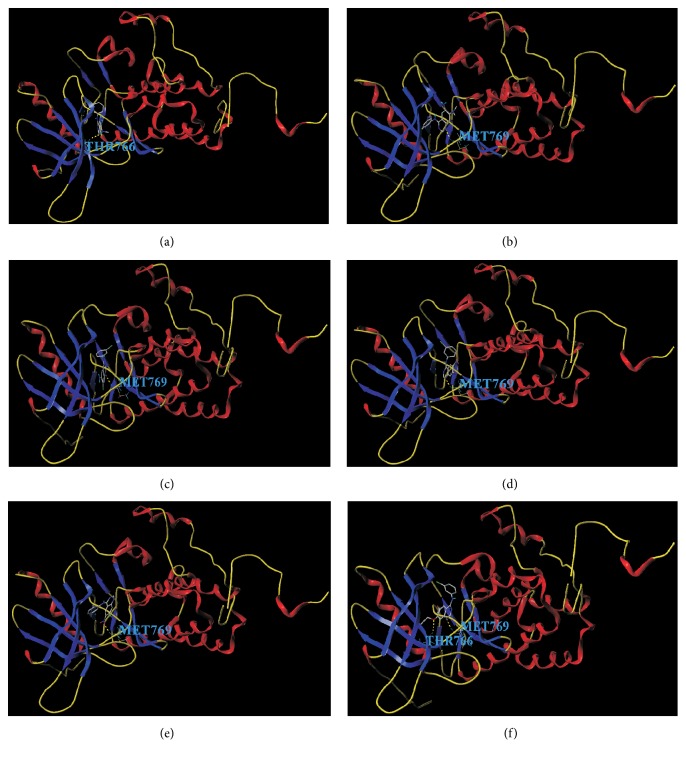
The docking result of the EGFR inhibitors with EGFR. (a) The binding site of compound** 27** with EGFR is Thr766. (b) The binding site of compound** 28** with EGFR is Met769. (c) The binding site of compound** 30** with EGFR is Met769. (d) The binding site of compound** 31** with EGFR is Met769. (e) The binding site of compound** 32** with EGFR is Met769. (f) The binding sites of compound** 33** with EGFR is Thr766 and Met769.

Compounds** 27**,** 28**,** 30**,** 31**,** 32,** and** 33** were used for molecular docking with EGFR. As a result, these compounds have hydrogen bonds at Thr766 and Met769 which were in ATP binding sites (see Figure 4). These compounds interact with EGFR kinase at binding sites and the quinolone ring bound to the hydrophobic pocket of EGFR, instead of the purine ring of ATP.

## 4. Discussion

### 4.1. 2D-QSAR Model

Feature selection via removal of some unnecessary features is required for a precise prediction model [25, 54, 55]. A subset containing nine features was obtained to build the 2D-QSAR prediction model. The prediction accuracy of the model was well for the training set and independent test set. This result indicated that the original data contained some redundant features, and feature selection was a helpful step in building a prediction model.

Although the accuracy of the prediction model with a subset containing nine features (DPLL, H, HF, HOMO, MR, Pc, TIndx, VP, and WIndx) was reliable, it was difficult to analyze the relationship between these descriptors and the activity of EGFR inhibitors as the prediction model is nonlinear. Thus, sensitivity was further applied for this problem [56].  Figure 1(a) shows the relationship between the Dipole length and activity. When the Dipole length is approximately 2 and 6.5, the activity levels are at minimum and maximum, respectively.  Figure 1(b) shows the relationship between Henry's law constant and activity. The activity increases along with Henry's law constant from 0 to 30. When Henry's law constant is more than 30, the activity has a rising trend.  Figure 1(c) shows the relationship between the Heat of Formation and activity. When the Heat of Formation ranges from −700 to 600, the activity increases. When the Heat of Formation is more than 600, the activity has a rising trend.  Figure 1(d) shows the relationship between the HOMO energy and activity. When the HOMO energy ranges from −9.25 to −8.25, the activity increases. When the HOMO energy is approximately −8.25, the activity peaks. When the HOMO energy is greater than −8.25, the activity decreases. When the HOMO energy is more than −7.25, the activity has a decreasing trend.  Figure 1(e) shows the relationship between the Molar refractivity and activity. When the Molar refractivity is approximately 10 and 14, the activity levels are at minimum and maximum, respectively.  Figure 1(f) shows the relationship between the critical pressure and activity. When the critical pressure ranges from 0 to 60, the activity increases. When the critical pressure is more than 60, the activity has a rising trend.  Figure 1(g) shows the relationship between the molecular topological index and activity. When the molecular topological index ranges from 0 to 60,000, the activity decreases. When the molecular topological index is more than 60,000, the activity has a decreasing trend.  Figure 1(h) shows the relationship between the Vapor pressure and activity. When the Vapor pressure ranges from 0 to 1.4, the activity decreases. When the Vapor pressure was more than 1.4, the activity had a decreasing trend.  Figure 1(i) shows the relationship between the Wiener index and activity. When the Wiener index and activity range from 0 to 9,000, the activity decreases. When the Wiener index is more than 9,000, the activity has a decreasing trend.

### 4.2. 3D-QSAR Model

Molecules in the topomer CoMFA models can be split into two, three, four, and more groups as needed [51, 57]. In this study, compounds were divided into two groups (R1 and R2). EGFR inhibitors' activity was related to the completeness of the pharmacophore. In topomer CoMFA models, the pharmacophore is related to cutting [44, 48, 58], which plays an important role in the model's predictive performance of the model [58]. In the topomer CoMFA analysis, all molecules of the training set are cut into two fragments. While the fragmentation was complete, the input structures were standardized and the topomers were generated. They all shared the same identical substructure. If the same identical substructure was recognized by the test set, the model's predictive ability was promising.

**Figure 5:**
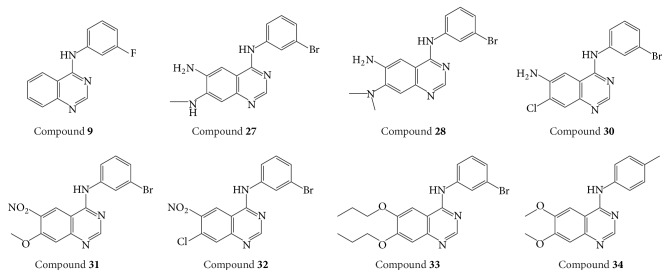
Structures of compounds** 9**,** 27**,** 28**,** 30**,** 31**,** 32**,** 33,** and** 34**.

It could be found that model 2 added an *N* element in R1 based on model 1, which contributed to the model's predictive ability (see Table 2). Thus, it is speculated that R1 and R2 in model 2 are the same identical substructures. The independent set test was used for evaluating model 2 (see Figure 2). It was observed that the predicted pIC_50_ of some compounds was poor, such as compound** 9** and compound** 34 **(see Table 3). We guess this is because the same identical substructures of the two compounds (see Figure 5) were different from the other compounds. The poor predicted pIC_50_ of compounds may cause high MAE. According to Roy et al.'s report [46], the 3D-QSAR model in our study was reliable as the MAE of the external validation was both less than 0.1 × training set range and MAE (training set) + 3 × *σ*. It is well known that the presence of systematic error in predictions may easily be identified from the difference in mean error and mean absolute error. It is important to analyze prediction errors of compounds in test set in order to search any possible systematic error. In Roy et al.'s study [59], various metrics, including the number of positive prediction errors (NPE), the number of negative prediction errors (NNP), the absolute value for average of prediction errors (AE), the average of absolute prediction errors (AAE), the mean of positive prediction errors (MPE), and the absolute value for mean of negative prediction errors (MNE), were employed to analyze the prediction's error. If prediction error is complied with principles I–V defined by Roy, the results were recommended. In our study, the NPE, NNP, AE, AAE, MPE, and MNE were 12, 11, 0.219, 0.526, 0.713, and −0.321, respectively. ABS (MPE/MNE) and *R*^2^ (*Y* versus residuals) were 2.2 (threshold = 2) and 0.67 (threshold = 0.5), respectively. Hence, it was regarded that our 3D-QSAR model is reliable.

In addition, topomer CoMFA model provides opinions on modifying EGFR inhibitors in order to design potential highly selective and highly active EGFR inhibitors. Compound** 33** (see Figure 5) was chosen to study the effect of R1 and R2 groups on activity due to its high activity. In R1 group, large group with a positive-charge in the yloxyethyl increases the compound's bioactivity (see Figure 3). In R2 group, small groups with a positive-charge in the benzene ring may also increase the compound's bioactivity.

### 4.3. Molecular Docking Analysis

Molecular docking was applied to predict the interaction sites between compounds and EGFR. As the structure of compound** 33** is similar to erlotinib, EGFR also interacts with compound** 33** at Thr766 and Met769 [50]. Interestingly, it is observed that the binding modes of compound 33-EGFR and erlotinib-EGFR were different despite the similar structure after calculation. Quinolone ring of erlotinib competitively binds to the hydrophobic pocket of EGFR kinase. For erlotinib, the aniline group reached into the pocket, and substituent groups of site 6 and site 7 were located outside of the hydrophobic pocket. For compound** 33**, it interacts with the EGFR by substituent groups of site 6 and site 7 in the hydrophobic pocket. In the steric and electrostatic fields, large volume group and positively charged group in site 6 and site 7 of compound** 33** may increase inhibitor activity (see Figure 3). Then, the similar chemical series of compound** 33** was selected to study the docking site. As a result, compounds** 28**,** 30**,** 31**, and** 32** interact with EGFR at Met769, and compound** 27** interacts with EGFR at Thr766. Thus, we considered that the Thr766 and Met769 played a crucial role in the EGFR activity.

Many studies performed the QSAR on kinase inhibitors, and the result was helpful for drugs design. In Farghaly et al.'s study [60], QSAR model was built, and the RMSE and *r*^2^ were applied to evaluate the model. After calculating, they selected out three predominant descriptors affecting the anticancer activity, and five anticancer agents were screened finally. Sharma showed the 2D-QSAR studies of c-Src tyrosine kinase inhibitors with *q*^2^ = 0.755 and *r*^2^ = 0.832 [61]. Sharma et al. reported QSAR studies of Aurora A kinase inhibitors [62]. *q*^2^ is 0.762 and *r*^2^ is 0.806. The difference in the number of samples causes the difference in *q*^2^ and *r*^2^. When *q*^2^ and *r*^2^ are more than 0.5 and 0.8, respectively, the model has statistical significance. In our QSAR study, *q*^2^ is 0.565 lower than these two studies, but *r*^2^ is higher (see Table 4). In addition, steric and electrostatic field and molecular docking analysis were applied in our study to explore the activity development and predict the interaction between inhibitors and protein, which is not showed in these studies. In conclusion, QSAR combined with molecular docking provides better insight into the future design of more potent EGFR inhibitors prior to synthesis.

## 5. Conclusion

In this study, 2D-QSAR and 3D-QSAR prediction models were built to analyze EGFR inhibitors. Firstly, the 2D-QSAR model was built to predict whether a compound was an inhibitor or a noninhibitor. The accuracy of the 2D-QSAR model using the tenfold cross-validation test and independent set test was 98.99% and 97.67%, respectively. Then, the topomer CoMFA model was built based on EGFR inhibitors. Two models were obtained by cutting different molecular bonds. As a result, model 2 with higher *q*^2^ value and *r*^2^ values was selected to predict EGFR inhibitors. Finally, a series of similar chemical inhibitors were selected to study the interacting sites between EGFR and EGFR inhibitors using molecular docking tool. As a result, Thr766 and Met769 were received by studying the docking result. Thus, we considered that Thr766 and Met769 played a crucial role in the EGFR activity.

## Supplementary Material

The Supplementary Material includes: 1. A training set for building prediction model. 2. A test set for validation.

## Figures and Tables

**Table 1:** The results of prediction accuracy for different data sets containing 9 molecular descriptors using SVM classifier. DS and EP present data set and evaluation parameters, respectively.

EP	DS			
	Train set (75%)	Train set (70%)	Train set (50%)	Test set (30%)
SN (%)	97.22	98.55	91.94	96.77
SP (%)	98.59	99.23	90.67	98.18
ACC (%)	98.13	98.99	91.24	97.67
MCC	0.958	0.978	0.824	0.950

**Table 2:** Results from two topomer CoMFA model studies.

Dataset	Topomer CoMFA model 1	Topomer CoMFA model 2
Cutting model	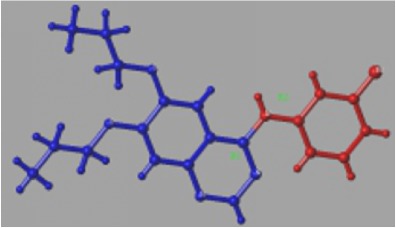	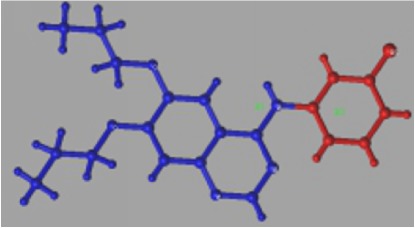
*q* ^2^	0.483	0.565
*r* ^2^	0.773	0.888

**Table 3:** Experimental and predicted PIC_50_ for topomer CoMFA model 2.

Compound	Exp	Pre
Training set		
2	7.64	6.62
4	6.24	6.2
5	6.04	6.45
7	6	6.16
8	8	8.15
10	7.25	7.05
11	6.11	6.62
13	7	6.31
15	6.09	6.06
16	6.26	6.14
17	7.53	8.02
18	9.5	9.06
20	8.39	8.28
22	7.92	8.01
23	8.32	7.59
24	8.15	8.05
25	7.92	8.22
26	7.95	7.78
27	9.16	8.64
29	8.42	8.87
30	8.18	8.3
31	7.82	8.03
32	7.6	7.26
33	9.76	9.6
34	9.01	8.05
36	8.11	7.94
37	7.74	7.43
38	7.35	7.31
40	8.01	8.59
41	8.36	8.46
42	7.45	7.71
43	7.88	7.7
45	6.6	6.36
46	7.39	7.84
47	8	7.5
48	7.04	6.87
50	6.88	6.82
51	6.17	6.08
53	5.74	6.36
54	5.31	5.72
55	6.07	7.21
56	6.92	7.4
57	7.39	6.9
58	7.29	7.14
60	6.9	7.15
61	8.58	8.47
63	6.16	5.85
64	6.02	6.36
65	7.28	6.86
66	6.48	6.54
67	6.58	7
69	7.08	7.57
70	8.82	8.38
71	9.11	8.97
72	9.02	8.97
73	8.42	8.96
75	8.53	9.2
76	8.63	8.35
77	6.42	6.97
78	7.76	7.78
79	8.36	8.34
80	8.63	8.39
81	6.19	6.8
82	8.52	7.97
83	8.05	8.04
85	7.1	7.16
86	7.5	7.57
87	7.26	7.52
88	6.04	6.06
90	4.33	4.35
91	4.66	4.62
92	5	5.52
94	7.19	7.17
95	6.23	5.89
97	4.14	3.98
98	8.05	7.54
99	6.97	6.79

Test set		
1	6.46	5.58
3	7.57	7.72
6	6.45	6.16
9	7.25	6.44
12	6.24	7.24
14	5.21	5.87
19	9.05	9.14
21	7.07	7.41
28	6.79	7.33
35	7.46	7.23
39	8.5	8.53
44	7.4	8.31
49	5.43	6.4
52	5.27	6.36
59	7.39	7.25
62	8.63	8.41
68	7.88	7.81
74	9.09	8.98
84	6.72	7.69
89	5.94	5.67
93	7.17	6.68
96	5.01	6.82
100	6.2	6.18

**Table 4:** The comparison of metrics between other studies and ours in QSAR study of the kinase inhibitors.

Metric	QSAR study		
	c-src tyrosine kinase inhibitors [61]	Aurora inhibitors [62]	Our study
*q* ^2^	0.755	0.762	0.565
*r* ^2^	0.832	0.806	0.888
